# Rodent islet amyloid polypeptide (IAPP) selectively enhances GABA_*A*_ receptor-mediated neuronal inhibition in mouse ventral but not dorsal hippocampal dentate gyrus granule cells

**DOI:** 10.3389/fncel.2025.1531790

**Published:** 2025-02-19

**Authors:** Olga Netsyk, Sergiy V. Korol, Gunilla T. Westermark, Bryndis Birnir, Zhe Jin

**Affiliations:** Department of Medical Cell Biology, Uppsala University, Uppsala, Sweden

**Keywords:** amylin, GABA_*A*_ receptor, hippocampus, islet amyloid polypeptide, neuronal inhibition, synaptic transmission, GABA

## Abstract

Islet amyloid polypeptide (IAPP, amylin) is a peptide hormone that plays an important role in glucose homeostasis but has been implicated in the pathophysiology of type 2 diabetes and Alzheimer’s disease. However, its effect on neurotransmission in the hippocampus remains poorly understood. Here, we investigated the impact of non-amyloidogenic rodent IAPP (rIAPP) on GABA_*A*_ receptor-mediated neuronal inhibition in mouse dorsal and ventral hippocampal dentate gyrus (DG) granule cells. Using whole-cell patch-clamp recordings, we showed that rIAPP selectively enhanced both GABA-activated spontaneous and miniature inhibitory postsynaptic currents (sIPSCs and mIPSCs) in ventral, but not dorsal, hippocampal DG granule cells. The effect of rIAPP on sIPSCs was completely abolished in the presence of the amylin receptor antagonist IAPP_8–37_. Interestingly, GABA_*A*_ receptor-mediated tonic current density remained unchanged in either dorsal or ventral hippocampal DG granule cells during rIAPP application. This region-specific and inhibition type-specific effect of rIAPP is likely associated with differential modulation of presynaptic GABA release as well as postsynaptic GABA_*A*_ receptors in the ventral as compared to the dorsal hippocampus. Our results suggest that rodent IAPP acts as a neuromodulator in hippocampal subregions by altering the strength of GABA_*A*_ receptor-mediated inhibitory signaling.

## Introduction

Metabolic hormones, including insulin, leptin, and glucagon-like peptide-1 (GLP-1), regulate glucose homeostasis and body metabolism. Additionally, mounting evidence suggests that these hormones and their analogs exert neuromodulatory effects in the central nervous system (Wang et al., 2024; Ferrari et al., 2022; Irving and Harvey, 2021; Hammoud et al., 2021; Jin et al., 2011b; Lathe, 2001). Islet amyloid polypeptide (IAPP), also known as amylin, is a peptide hormone co-released with insulin from pancreatic β cells. In addition to its peripheral effect on glucose regulation and energy homeostasis (Boyle et al., 2022), IAPP also exerts substantial effects on the central nervous system (Foll and Lutz, 2020). The plasma IAPP level is typically measured in the picomolar range, although it indeed varies depending on factors such as fasting state, meal consumption and diseases (Hartter et al., 1991; Phillips et al., 2001; Paulsson et al., 2014). IAPP efficiently crosses the blood-brain barrier (BBB) (Banks et al., 1995), and interacts with amylin receptors distributed throughout various brain regions, including the hindbrain, hypothalamus, and hippocampus (Becskei et al., 2004; Kimura et al., 2012). Amylin receptors (AMY1-3) are heterodimers composed of the calcitonin receptor (CTR) and one of the three receptor activity-modifying proteins (RAMP1-3), triggering intracellular signaling cascades that involve the Erk1/2 and Akt pathways (Fu et al., 2017a). It has been clearly demonstrated that IAPP signaling in the brain is associated with food intake, stress response, rewarding behavior and cognitive function (Geisler et al., 2024; Laugero et al., 2022; Foll and Lutz, 2020; Boccia et al., 2020). Notably, IAPP has been shown to contribute to the pathophysiology of Alzheimer’s disease (AD) (Corrigan et al., 2022; Zhang and Song, 2017; Oskarsson et al., 2015).

The hippocampus is an elongated curved structure in the medial temporal lobe, composed of several subregions, including cornu ammonis (CA) fields and the dentate gyrus (DG). DG acts as a gate or filter, regulating the flow of information from the entorhinal cortex to the CA3 regions. These subregions have distinct anatomical organization, electrophysiological properties, connectivity patterns and functional roles, which vary along hippocampal longitudinal axis (dorsal-ventral in rodents and posterior-anterior in primates) (Papatheodoropoulos, 2018; Strange et al., 2014; Risold and Swanson, 1996). The optimal hippocampal function depends on a fine-tuned balance between excitatory and inhibitory neurotransmission. Gamma-aminobutyric acid (GABA) plays a crucial role in maintaining inhibitory tone, which is manifested through both synaptic and extrasynaptic mechanisms (Farrant and Nusser, 2005). The activation of synaptic and extrasynaptic GABA_*A*_ receptors mediates inhibitory postsynaptic currents (IPSCs) and extrasynaptic currents, respectively (Sallard et al., 2021; Jin et al., 2011a; Birnir et al., 1994). Various molecules, including metabolic hormones such as insulin and GLP-1, can modulate these currents through direct and indirect mechanisms (Hammoud et al., 2021; Korol et al., 2015; Jin et al., 2011b; Wan et al., 1997). To date, the ability of IAPP to regulate GABA-activated inhibitory currents in hippocampal neurons remains unexplored.

Human IAPP was initially discovered in amyloid isolated from pancreatic tumors and islets from patients with type 2 diabetes (Cooper et al., 1987; Westermark et al., 1986), indicating its amyloidogenic nature. In contrast, the mouse and rat IAPP is non-amyloidogenic and differ from human IAPP in six positions. Especially, the proline residues at positions 25, 28, and 29 protect rodent IAPP against the formation of cytotoxic amyloid (Fortin and Benoit-Biancamano, 2015; Hay et al., 2015). In this study, we sought to examine whether the application of rodent IAPP at a physiologically relevant concentration (in the picomolar range) could modulate GABA_*A*_ receptor-mediated synaptic and extrasynaptic tonic currents in DG granule cells of mouse dorsal and ventral hippocampus, and if so, are such effects reversed by amylin/IAPP receptor antagonist.

## Materials and methods

### Animals

Male mice (C57BL/6J) between 8 and 10 weeks of age were used in this study. All experimental procedures were conducted following local ethical guidelines and protocols, approved by Uppsala Animal Ethical Committee, Swedish law and regulations based on Directive 2010/63/EU, C129/14.

### Hippocampal slice preparation

The preparation of hippocampal slices followed the previously described protocols (Jin et al., 2011a; Netsyk et al., 2020; Ting et al., 2014). In brief, the brain was swiftly removed and immersed in an ice-cold N-methyl D-glucamine (NMDG)-based cutting solution, which comprised of (in mM): 93 NMDG, 2.5 KCl, 1.2 NaH_2_PO_4_, 30 NaHCO_3_, 20 HEPES, 25 D-glucose, 10 MgSO_4_, 0.5 CaCl_2_, 5 sodium ascorbate, 2 thiourea, and 3 sodium pyruvate, pH 7.3–7.4 when saturated with 95% O_2_ and 5% CO_2_ and with an osmolarity of 300–305 mOsm. Brain slices (350 μm) were obtained using Leica VT 1200S microtome (Leica Microsystems AB, Germany). Dorsal and ventral hippocampal slices prepared as previously described (Hammoud et al., 2021; Netsyk et al., 2020) were initially placed in NMDG-based solution for 12–15 min at 32°C, followed by transferring to a HEPES-based holding solution containing (in mM): 92 NaCl, 2.5 KCl, 1.2 NaH_2_PO_4_, 30 NaHCO_3_, 20 HEPES, 25 D-glucose, 2 MgSO_4_, 2 CaCl_2_, 5 sodium ascorbate, 2 thiourea, and 3 sodium pyruvate, pH 7.3–7.4 and osmolarity 300–305 mOsm. All slices were maintained in this holding solution at room temperature (20–22°C) for a minimum of 1 h before recording.

### Electrophysiology

Whole-cell patch-clamp recordings were conducted on DG granule cells from both dorsal and ventral hippocampal slices. All experiments were performed at room temperature. Slices were transferred to a recording chamber and perfused with artificial cerebrospinal fluid (ACSF) containing (in mM): 119 NaCl, 2.5 KCl, 1.3 MgSO_4_, 1 NaH_2_PO_4_, 26.2 NaHCO_3_, 2.5 CaCl_2_, 11 D-glucose, and 3 kynurenic acid (to block glutamatergic synaptic transmission), pH 7.3–7.4 and osmolarity 300–305 mOsm, equilibrated with 95% O_2_ and 5% CO_2_. The resistance of glass patch pipettes was 3.4–4 MΩ when filled with an intracellular solution comprising (in mM): 140 CsCl, 8 NaCl, 2 EGTA, 0.2 MgCl_2_, 10 HEPES, 2 MgATP, 0.3 Na_3_GTP, and 5 QX314-Br, pH 7.2 and osmolarity 285–290 mOsm. Voltage-clamp recordings were conducted at the holding potential of −60 mV with a sampling rate of 10 kHz and filtered at 2 kHz using a Multipatch 700B amplifier, and digitized with an Axon Digidata board 1550A, which were controlled by pCLAMP 10.5 software (Axon Instruments, Molecular Devices). GABA-activated spontaneous inhibitory postsynaptic currents (sIPSCs) were recorded for a minimum of 5 min after baseline stabilization, and miniature IPSCs (mIPSCs) recordings were performed in the presence of tetrodotoxin (TTX, 1 μM). GABA_*A*_R antagonist picrotoxin (100 μM) was applied at the end of each recording to reveal the extrasynaptic GABA_*A*_R-mediated extrasynaptic tonic currents.

All chemicals and drugs were obtained from Sigma-Aldrich (Steinheim, Germany), except for rodent IAPP (amylin, Cat. No. 4030201) and amylin receptor antagonist amylin (8–37) (mouse, rat, Cat. No. 4030346) from BACHEM Ltd (Bubendorf, Switzerland), and TTX from Alomone Labs Ltd (Jerusalem, Israel).

### Data analysis

Recordings were rejected for analysis if the serial resistance varied by more than 20%. Analysis of sIPSCs and mIPSCs was conducted using MiniAnalysis software 6.0 (Synaptosoft, USA). IPSCs were identified as events based on a threshold of 5× the root-mean-square (RMS) of baseline noise, followed by visual inspection to eliminate false-positives. Only single-peak IPSC events were included in amplitude and kinetics analysis (Hammoud et al., 2021; Netsyk et al., 2020). Extrasynaptic tonic current amplitude was measured as a baseline shift in holding current after applying a GABA_*A*_R inhibitor picrotoxin (PTX). The IPSC charge transfer Q (fC) was measured from integral area that is determined by amplitude, rise and decay time in MiniAnalysis. The total IPSC current was calculated as frequency (s^–1^) × Q (fC). Synaptic and extrasynaptic current densities were calculated by normalizing total currents to cell membrane capacitance (Cm) (Netsyk et al., 2020).

### Statistical analysis

All data were analyzed with GraphPad Prism 10 (GraphPad Software, La Jolla, USA). Each group data was tested for normality with Shapiro–Wilk test. Paired Student *t*-test or Wilcoxon matched-pairs signed rank test was performed based on the data distribution. Statistical significance was defined as *P* < 0.05.

## Results

### Rodent IAPP potentiates GABA-activated spontaneous IPSCs (sIPSCs) in DG granule cells in ventral but not dorsal mouse hippocampus

**FIGURE 1:**
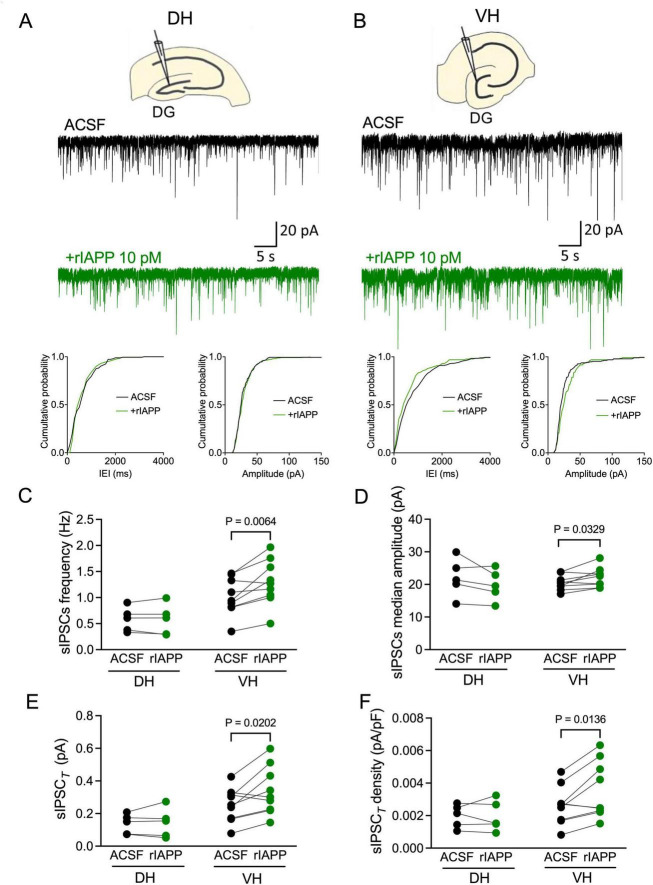
Rodent islet amyloid polypeptide (rIAPP) potentiated spontaneous synaptic GABA_A_ receptor-mediated currents in the dentate gyrus granule cells in ventral, but not in dorsal mouse hippocampus. **(A,B)** Schematic illustrations and representative traces of spontaneous inhibitory postsynaptic currents (sIPSCs) recorded from DG granule cells in dorsal [DH, **(A)**] and ventral [VH, **(B)**] hippocampus under control conditions (ACSF, black trace) and bath rIAPP (10 pM) application (green trace). Cumulative probability plots of the inter-event interval (IEI) and median amplitude of sIPSCs from the above representative traces. Summary statistics of frequency **(C)**, median amplitude **(D)**, total synaptic currents (sIPSC*_*T*_*) **(E)** and total current density [sIPSC*_*T*_* density, **(F)**] of sIPSC recorded from DG granule cells of DH (*n* = 5) and VH (*n* = 9) under control (ACSF, black color) and 10 pM rIAPP application (green color). Data are presented as scatter dot plots for individual values with connected lines, indicating data obtained from individual cells before (black) and during rIAPP application (green). The paired Student’s *t*-test was used for statistical analysis. V_hold_ = –60 mV.

To examine whether rIAPP affects GABA-activated inhibitory currents along the hippocampal longitudinal axis, we recorded sIPSCs in DG granule cells from dorsal and ventral hippocampal slices and acutely applied rIAPP (10 pM) for minimal 15 min. Representative sIPSC recordings in dorsal and ventral hippocampus are shown in Figures 1A, B and Supplementary Figures 1A, B, respectively. The mean frequency, median amplitude, total synaptic current, and current density of sIPSCs were significantly increased after rIAPP application in DG granule cells from ventral hippocampus (Figures 1C–F), whereas rise time (10–90%), decay time (63%), and median charge transfer remained unchanged (Supplementary Table 1). In contrast, in dorsal hippocampus rIAPP did not alter any sIPSC parameters in recorded DG granule cells (Figures 1C–F and Supplementary Table 1). These results show that rIAPP selectively enhances GABA-activated synaptic currents in hippocampal subregions.

We further examine whether the sIPSC enhancement by rIAPP is via amylin receptors in the ventral hippocampus. An amylin receptor antagonist IAPP_8–37_ (1 μM), was initially perfused in the recording chamber, followed by co-perfusion of rIAPP and IAPP_8–37_ (Supplementary Figure 2A). In ventral hippocampal DG granule cells in the presence of IAPP_8–37_, rIAPP no longer enhanced the sIPSC frequency and median amplitude (Supplementary Figures 2B, C). Thus, blocking the amylin receptor abolished the rIAPP-induced potentiation of the sIPSCs in ventral hippocampal DG granule cells.

### rIAPP increases GABA-activated miniature IPSCs (mIPSCs) total current density in ventral but not dorsal hippocampal DG granule cells

**FIGURE 2:**
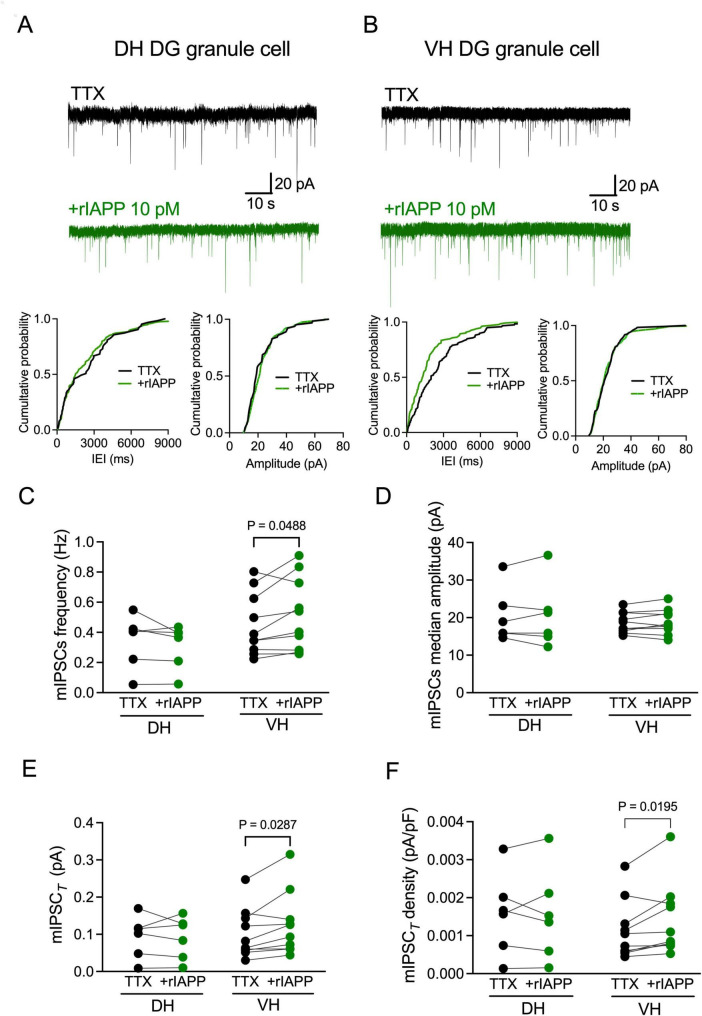
Rodent IAPP potentiated miniature GABA_A_ receptor-mediated currents in the dentate gyrus granule cells in ventral mouse hippocampus. **(A,B)** Representative traces of miniature inhibitory postsynaptic currents (mIPSCs) recorded from DG granule cells in dorsal [DH, **(A)**] and ventral [VH, **(B)**] hippocampus before (black trace) and during rIAPP (10 pM) application (green trace) in the constant presence of tetrodotoxin (TTX, 1 μM). Cumulative probability plots of the inter-event interval (IEI) and median amplitude of mIPSCs from the above representative traces. **(C–F)** Summary statistics of frequency **(C)**, median amplitude **(D)**, total synaptic current [mIPSC*_*T*_*, **(E)**] and total current density [mIPSC*_*T*_* density, **(F)**] of mIPSCs recorded from DG granule cells of DH (*n* = 6 cells) and VH (*n* = 10 cells) under control (TTX, black color) and 10 pM rIAPP application (green color). Data are presented as scatter dot plot for individual values with connected lines, indicating data obtained from individual cells before (black) and during rIAPP application (green). The paired Student’s *t*-test or Wilcoxon matched-pairs signed rank test was used for statistical analysis. V_hold_ = –60 mV.

The augmentation of the sIPSCs by rIAPP can be associated with pre- or post-synaptic mechanisms. We thus examined the effect of rIAPP on mIPSCs in the presence of a voltage-gated sodium channel blocker TTX (1 μM), which eliminates action potential-dependent GABA release. Representative current traces are shown for the dorsal (Figure 2A and Supplementary Figure 3A) and ventral (Figure 2B and Supplementary Figure 3B) hippocampal DG granule cells. rIAPP significantly increased mIPSC frequency (Figure 2C), median charge transfer (Supplementary Table 1), total synaptic current (Figure 2E), and total current density (Figure 2F) but not median amplitude (Figure 2D), rise time (10–90%) or decay time (63%) (Supplementary Table 1), in the DG granule cells of ventral hippocampus. In contrast, rIAPP had no effects on mIPSCs recorded in the dorsal hippocampal DG granule cells (Figures 2A, C–F and Supplementary Table 1), which was similar to the observations for the sIPSCs (Figures 1A, C–F). These data indicate that rIAPP affects pre-synaptic GABA release only in the ventral hippocampal DG granule cells.

### GABA_*A*_R-mediated extrasynaptic tonic current is not altered by rIAPP in mouse hippocampal DG granule cells

**FIGURE 3:**
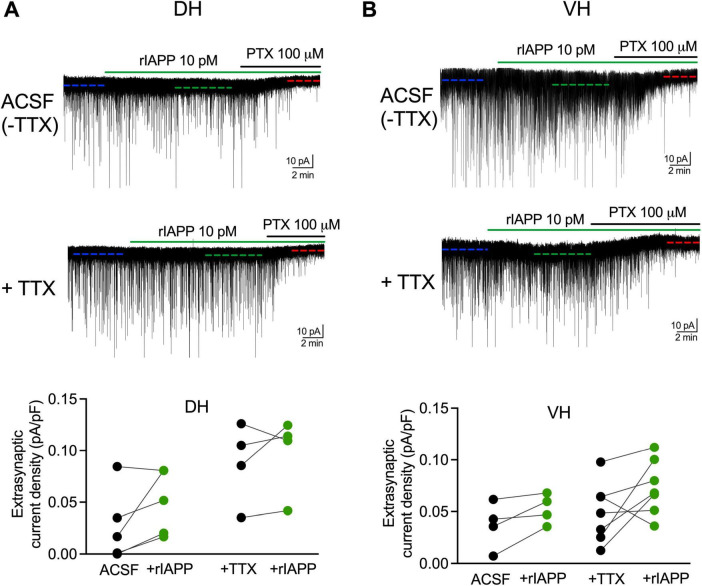
Rodent IAPP did not affect GABA_*A*_R-mediated extrasynaptic current in DG granule cells along hippocampal dorsal-ventral axis. Representative GABA_*A*_R-mediated current traces and summary statistics of extrasynaptic current density in dentate granule cells of DH **(A)** and VH **(B)** recorded in ACSF without TTX or constant presence of tetrodotoxin (+TTX, 1 μM) with rIAPP (10 pM) application (green horizontal line). The extrasynaptic current was revealed by the application of GABA_*A*_R antagonist picrotoxin (PTX, 100 μM, black horizontal line) and its current amplitude was calculated by the shift of the baseline current (dashed line) under different conditions. Data are presented as scatter dot plots for individual values with connected lines, indicating data obtained from individual cells before (black) and during rIAPP application (green). *n* = 4–7 cells. The paired Student’s *t*-test was used for statistical analysis. V_hold_ = –60 mV.

GABA-activated neuronal inhibition consists of not only synaptic but also extrasynaptic components, each with distinct properties and functional roles. Therefore, we further examined whether rIAPP changed the GABA_*A*_R-mediated extrasynaptic tonic currents in the absence or presence of TTX. The GABA tonic current was revealed as a shift of baseline current by applying the GABA_*A*_R antagonist PTX (Figures 3A, B). The extrasynaptic current density was not changed by rIAPP (10 pM) in DG granule cells in either dorsal (Figure 3A) or ventral (Figure 3B) hippocampus in the absence or presence of TTX. These results suggest rIAPP does not influence GABA-activated extrasynaptic tonic inhibition in mouse hippocampal DG granule cells.

## Discussion

**FIGURE 4:**
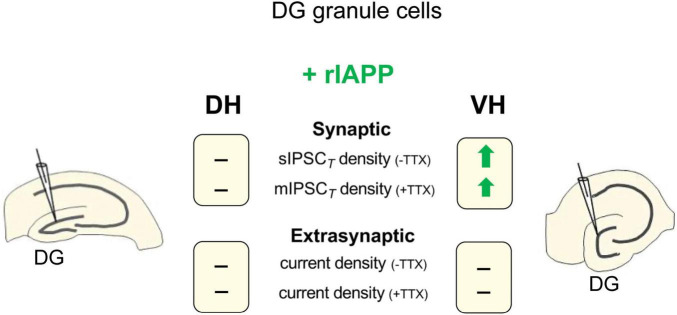
Schematic summary of rIAPP effects on GABA_*A*_R-mediated synaptic and extrasynaptic current density in dentate gyrus (DG) granule cells of mouse dorsal (DH) and ventral (VH) hippocampus. -TTX, in the absence of TTX; +TTX, in the presence of TTX; —, no change; 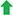, current density increased by rIAPP (10 pM).

A substantial body of research has provided evidence that the hippocampus is not homogenous and has distinct characteristics and functions, especially in the dorsal and ventral subregions (Netsyk et al., 2020; Papatheodoropoulos, 2018; Strange et al., 2014; Risold and Swanson, 1996). Our findings demonstrate that rIAPP affects GABA_A_ receptor-mediated currents in the mouse hippocampus in both a region-specific and inhibition type-specific manner. Specifically, we have observed that rIAPP selectively potentiates synaptic GABAergic transmission in the ventral, but not the dorsal, hippocampal DG granule cells (Figure 4). Our results expand our understanding of how metabolic hormones influence GABAergic inhibition in the hippocampus (Hammoud et al., 2021; Korol et al., 2015; Jin et al., 2011b; Solovyova et al., 2009).

Hippocampal DG granule cells exhibit distinctively low levels of excitability, which is mainly attributed to the extensive GABAergic inhibition present in DG (Coulter and Carlson, 2007). Notably, a diverse array of synaptic and extrasynaptic GABA_A_ receptors are expressed in DG granule cells, mediating IPSCs and tonic inhibitory currents when activated by GABA (Birnir et al., 1994). Our results reveal that rIAPP enhances sIPSCs exclusively in ventral hippocampal DG granule cells, whereas insulin (1 nM), as shown in our previous study, potentiates sIPSCs in both dorsal and ventral regions (Hammoud et al., 2021). rIAPP potentiates sIPSCs in ventral hippocampal DG granule cells, as evidenced by increased frequency and amplitude, suggesting both pre- and post-synaptic mechanisms. In contrast, insulin selectively increases the frequency, but not the amplitude of sIPSCs in these cells from 8 to 10 weeks old wild-type mice. However, this insulin effect is absent in 5–6 months old wild-type or AD tg-APPSwe mice, indicating an age- and disease-dependent modulation (Hammoud et al., 2021). The increased frequency indicates a potential enhancement of pre-synaptic GABA release probability from GABAergic interneurons, while the increased amplitude might reflect both pre- and post-synaptic changes, such as increased GABA concentration per vesicle released (Yamashita et al., 2018; Takamori, 2016) or increased GABA_A_ receptor density or conductance (Netsyk et al., 2020). In the presence of TTX, rIAPP-induced increase in mIPSC frequency and total current density, without changes in amplitude, suggests that rIAPP mainly affects pre-synaptic GABA release only in ventral hippocampal DG granule cells. The potentiated pre-synaptic GABA release may involve increased GABA concentration per vesicle released, increased probability of GABA vesicle release, increased size or mobility of the readily releasable pool of GABA vesicles, interactions with pre-synaptic auto-receptors or GABA transporters. Similarly, insulin also enhances mIPSC only in ventral DG granule cells, primarily via a presynaptic mechanism (Hammoud et al., 2021). In contrast, rIAPP did not change extrasynaptic GABA_A_ receptor-mediated tonic currents in hippocampal DG granule cells. This finding suggests that rIAPP does not alter interstitial GABA concentration or extrasynaptic GABA_*A*_R. In summary, our findings reveal that rIAPP exerts selective modulation on various modes of GABAergic transmission in DG granule neurons.

The amylin receptor antagonist IAPP_8–37_ completely abolishes the rIAPP-induced potentiation of sIPSCs in ventral DG granule cells, confirming that this effect is mediated specifically through amylin receptors. The combination of CTR with different RAMPs (RAMP1, 2, 3), resulting in three amylin receptor subtypes (AMY1, AMY2 and AMY3) that bind IAPP/amylin with various affinities (Poyner et al., 2002). In heterozygous CRT knockout mice, the uptake of the amylin receptor antagonist cyclic AC253 is markedly reduced in the hippocampus (Soudy et al., 2017). Additionally, amylin-induced c-fos (a marker for neuronal activation) activity in the dentate gyrus is significantly decreased in RAMP1/3 knockout mice (Skovbjerg et al., 2021). These findings indicate the presence of functional AMY1 and AMY3 receptors in the hippocampal neurons. Interestingly, AMY3 is expressed not only in neurons but also in microglia and mediates Aβ-induced brain inflammation in a transgenic mouse model of AD (Fu et al., 2017b). However, the difference in distribution, cell type specificity and expression levels of AMY1 and AMY3 between the dorsal and ventral hippocampus have not yet been thoroughly investigated. Activation of amylin receptors increases intracellular cAMP levels, stimulating protein kinase A (PKA) and downstream signaling pathways (Fu et al., 2017a), which potentially alter pre-synaptic GABA release (Trudeau et al., 1996; Stephens, 2009). A recent study has demonstrated that activation of amylin receptors expressed on pre-synaptic GABAergic neurons in the ventral tegmental area enhances local GABA release, which subsequently inhibits the activity of projecting dopamine neurons (Geisler et al., 2024). Similarly, our findings clearly demonstrate an association between IAPP signaling and GABAergic transmission in the ventral hippocampus.

The regional specificity of rIAPP’s effects, confined to the ventral hippocampus, is particularly intriguing. This specificity may reflect regional differences between the dorsal and ventral hippocampus in the expression and distribution of amylin receptors, GABA_A_ receptors, and their downstream signaling pathways (Hammoud et al., 2021; Netsyk et al., 2020; Papatheodoropoulos, 2018; Strange et al., 2014). Given that the ventral hippocampus is more involved in emotion, stress response and metabolic control due to its distinct connectivity with various brain regions including the amygdala (Strange et al., 2014; Lathe, 2001; Risold and Swanson, 1996; Fanselow and Dong, 2010; Boccia et al., 2020), our findings suggest that rIAPP might modulate these functions through its region-specific enhancement of GABAergic inhibition. Future studies should investigate serval important questions, including the potential differences in amylin receptor expression patterns between the dorsal and ventral hippocampus, as well as examine how human IAPP and its non-amyloidogenic analogs, such as pramlintide, modulate GABAegic inhibition in these distinct hippocampal regions.

In conclusion, our results reveal a neuromodulatory role for rIAPP in the hippocampus, specifically enhancing GABAergic synaptic inhibition in the ventral region mainly through pre-synaptic mechanism. These findings provide new insights into the complex functions of IAPP/amylin in the brain and highlight the importance of considering regional heterogeneity in the hippocampus when studying its effects on neuronal function.

## Data Availability

The raw data supporting the conclusions of this article will be made available by the authors, without undue reservation.

## References

[B1] Banks W. A. Kastin A. J. Maness L. M. Huang W. Jaspan J. B. (1995). Permeability of the blood-brain barrier to amylin. *Life Sci.* 57 1993–2001. 7475950 10.1016/0024-3205(95)02197-q

[B2] Becskei C. Riediger T. Zund D. Wookey P. Lutz T. A. (2004). Immunohistochemical mapping of calcitonin receptors in the adult rat brain. *Brain Res.* 1030 221–233. 10.1016/j.brainres.2004.10.012 15571671

[B3] Birnir B. Everitt A. B. Gage P. W. (1994). Characteristics of GABAA channels in rat dentate gyrus. *J. Membr. Biol.* 142 93–102. 7707357 10.1007/BF00233386

[B4] Boccia L. Gamakharia S. Coester B. Whiting L. Lutz T. A. Le Foll C. (2020). Amylin brain circuitry. *Peptides* 132:170366. 10.1016/j.peptides.2020.170366 32634450

[B5] Boyle C. N. Zheng Y. Lutz T. A. (2022). Mediators of amylin action in metabolic control. *J. Clin. Med.* 11:2207. 10.3390/jcm11082207 PMC9025724 35456307

[B6] Cooper G. J. Willis A. C. Clark A. Turner R. C. Sim R. B. Reid K. B. (1987). Purification and characterization of a peptide from amyloid-rich pancreases of type 2 diabetic patients. *Proc. Natl. Acad. Sci. U.S.A.* 84 8628–8632. 10.1073/pnas.84.23.8628 3317417 PMC299599

[B7] Corrigan R. R. Piontkivska H. Casadesus G. (2022). Amylin pharmacology in Alzheimer’s disease pathogenesis and treatment. *Curr. Neuropharmacol.* 20 1894–1907. 34852745 10.2174/1570159X19666211201093147 PMC9886804

[B8] Coulter D. A. Carlson G. C. (2007). Functional regulation of the dentate gyrus by GABA-mediated inhibition. *Prog. Brain Res.* 163 235–243. 17765722 10.1016/S0079-6123(07)63014-3

[B9] Fanselow M. S. Dong H. W. (2010). Are the dorsal and ventral hippocampus functionally distinct structures? *Neuron* 65 7–19. 20152109 10.1016/j.neuron.2009.11.031 PMC2822727

[B10] Farrant M. Nusser Z. (2005). Variations on an inhibitory theme: phasic and tonic activation of GABA(A) receptors. *Nat. Rev. Neurosci.* 6 215–229. 10.1038/nrn1625 15738957

[B11] Ferrari F. Moretti A. Villa R. F. (2022). Incretin-based drugs as potential therapy for neurodegenerative diseases: current status and perspectives. *Pharmacol. Ther.* 239:108277. 10.1016/j.pharmthera.2022.108277 36064147

[B12] Foll C. L. Lutz T. A. (2020). Systemic and central amylin, amylin receptor signaling, and their physiological and pathophysiological roles in metabolism. *Compr. Physiol.* 10 811–837. 10.1002/cphy.c190034 32941692

[B13] Fortin J. S. Benoit-Biancamano M. O. (2015). Wildlife sequences of islet amyloid polypeptide (IAPP) identify critical species variants for fibrillization. *Amyloid* 22 194–202. 10.3109/13506129.2015.1070824 26300107

[B14] Fu W. Patel A. Kimura R. Soudy R. Jhamandas J. H. (2017a). Amylin receptor: a potential therapeutic target for Alzheimer’s disease. *Trends Mol. Med.* 23 709–720. 28694141 10.1016/j.molmed.2017.06.003

[B15] Fu W. Vukojevic V. Patel A. Soudy R. Mactavish D. Westaway D. (2017b). Role of microglial amylin receptors in mediating beta amyloid (Abeta)-induced inflammation. *J. Neuroinflamm.* 14:199. 10.1186/s12974-017-0972-9 28985759 PMC5639602

[B16] Geisler C. E. Decarie-Spain L. Loh M. K. Trumbauer W. Gaisinsky J. Klug M. E. (2024). Amylin modulates a ventral tegmental area-to-medial prefrontal cortex circuit to suppress food intake and impulsive food-directed behavior. *Biol. Psychiatry* 95 938–950. 10.1016/j.biopsych.2023.07.011 37517705 PMC13005266

[B17] Hammoud H. Netsyk O. Tafreshiha A. S. Korol S. V. Jin Z. Li J. P. (2021). Insulin differentially modulates GABA signalling in hippocampal neurons and, in an age-dependent manner, normalizes GABA-activated currents in the tg-APPSwe mouse model of Alzheimer’s disease. *Acta Physiol. (Oxf)* 232:e13623. 10.1111/apha.13623 33559388

[B18] Hartter E. Svoboda T. Ludvik B. Schuller M. Lell B. Kuenburg E. (1991). Basal and stimulated plasma levels of pancreatic amylin indicate its co-secretion with insulin in humans. *Diabetologia* 34 52–54. 10.1007/BF00404025 2055340

[B19] Hay D. L. Chen S. Lutz T. A. Parkes D. G. Roth J. D. (2015). Amylin: pharmacology, physiology, and clinical potential. *Pharmacol. Rev.* 67 564–600. 26071095 10.1124/pr.115.010629

[B20] Irving A. Harvey J. (2021). Regulation of hippocampal synaptic function by the metabolic hormone leptin: implications for health and disease. *Prog. Lipid Res.* 82:101098. 10.1016/j.plipres.2021.101098 33895229

[B21] Jin Z. Jin Y. Birnir B. (2011a). GABA-activated single-channel and tonic currents in rat brain slices. *J. Vis. Exp*. 17:2858. 10.3791/2858 21788935 PMC3196182

[B22] Jin Z. Jin Y. Kumar-Mendu S. Degerman E. Groop L. Birnir B. (2011b). Insulin reduces neuronal excitability by turning on GABA(A) channels that generate tonic current. *PLoS One* 6:e16188. 10.1371/journal.pone.0016188 21264261 PMC3021545

[B23] Kimura R. Mactavish D. Yang J. Westaway D. Jhamandas J. H. (2012). Beta amyloid-induced depression of hippocampal long-term potentiation is mediated through the amylin receptor. *J. Neurosci.* 32 17401–17406. 10.1523/JNEUROSCI.3028-12.2012 23197731 PMC6621862

[B24] Korol S. V. Jin Z. Babateen O. Birnir B. (2015). GLP-1 and exendin-4 transiently enhance GABAA receptor-mediated synaptic and tonic currents in rat hippocampal CA3 pyramidal neurons. *Diabetes* 64 79–89. 25114295 10.2337/db14-0668

[B25] Lathe R. (2001). Hormones and the hippocampus. *J. Endocrinol.* 169 205–231. 11312139 10.1677/joe.0.1690205

[B26] Laugero K. D. Tryon M. Mack C. Caldarone B. J. Hanania T. Mcgonigle P. (2022). Peripherally administered amylin inhibits stress-like behaviors and enhances cognitive performance. *Physiol. Behav.* 244:113668. 10.1016/j.physbeh.2021.113668 34863999

[B27] Netsyk O. Hammoud H. Korol S. V. Jin Z. Tafreshiha A. S. Birnir B. (2020). Tonic GABA-activated synaptic and extrasynaptic currents in dentate gyrus granule cells and CA3 pyramidal neurons along the mouse hippocampal dorsoventral axis. *Hippocampus* 30 1146–1157. 10.1002/hipo.23245 32533811

[B28] Oskarsson M. E. Paulsson J. F. Schultz S. W. Ingelsson M. Westermark P. Westermark G. T. (2015). In vivo seeding and cross-seeding of localized amyloidosis: a molecular link between type 2 diabetes and Alzheimer disease. *Am. J. Pathol.* 185 834–846. 10.1016/j.ajpath.2014.11.016 25700985

[B29] Papatheodoropoulos C. (2018). Electrophysiological evidence for long-axis intrinsic diversification of the hippocampus. *Front. Biosci. (Landmark Ed)* 23 109–145. 10.2741/4584 28930540

[B30] Paulsson J. F. Ludvigsson J. Carlsson A. Casas R. Forsander G. Ivarsson S. A. (2014). High plasma levels of islet amyloid polypeptide in young with new-onset of type 1 diabetes mellitus. *PLoS One* 9:e93053. 10.1371/journal.pone.0093053 24671002 PMC3966843

[B31] Phillips A. R. Abu-Zidan F. M. Farrant G. J. Zwi J. L. Cooper G. J. Windsor J. A. (2001). Plasma amylin concentration is related to the severity of intestinal ischemic injury in rats. *Surgery* 129 730–735. 11391372 10.1067/msy.2001.113892

[B32] Poyner D. R. Sexton P. M. Marshall I. Smith D. M. Quirion R. Born W. (2002). International Union of Pharmacology. XXXII. The mammalian calcitonin gene-related peptides, adrenomedullin, amylin, and calcitonin receptors. *Pharmacol. Rev.* 54 233–246. 10.1124/pr.54.2.233 12037140

[B33] Risold P. Y. Swanson L. W. (1996). Structural evidence for functional domains in the rat hippocampus. *Science* 272 1484–1486. 8633241 10.1126/science.272.5267.1484

[B34] Sallard E. Letourneur D. Legendre P. (2021). Electrophysiology of ionotropic GABA receptors. *Cell Mol. Life Sci.* 78 5341–5370. 34061215 10.1007/s00018-021-03846-2 PMC8257536

[B35] Skovbjerg G. Roostalu U. Hansen H. H. Lutz T. A. Le Foll C. Salinas C. G. (2021). Whole-brain mapping of amylin-induced neuronal activity in receptor activity-modifying protein 1/3 knockout mice. *Eur. J. Neurosci.* [Epub ahead of print]. 10.1111/ejn.15254 33905587

[B36] Solovyova N. Moult P. R. Milojkovic B. Lambert J. J. Harvey J. (2009). Bi-directional modulation of fast inhibitory synaptic transmission by leptin. *J. Neurochem.* 108 190–201. 10.1111/j.1471-4159.2008.05751.x 19094063 PMC2605943

[B37] Soudy R. Patel A. Fu W. Kaur K. Mactavish D. Westaway D. (2017). Cyclic AC253, a novel amylin receptor antagonist, improves cognitive deficits in a mouse model of Alzheimer’s disease. *Alzheimers Dement. (N Y)* 3 44–56. 10.1016/j.trci.2016.11.005 29067318 PMC5651374

[B38] Stephens G. J. (2009). G-protein-coupled-receptor-mediated presynaptic inhibition in the cerebellum. *Trends Pharmacol. Sci.* 30 421–430. 10.1016/j.tips.2009.05.008 19632729

[B39] Strange B. A. Witter M. P. Lein E. S. Moser E. I. (2014). Functional organization of the hippocampal longitudinal axis. *Nat. Rev. Neurosci.* 15 655–669. 25234264 10.1038/nrn3785

[B40] Takamori S. (2016). Presynaptic molecular determinants of quantal size. *Front. Synaptic Neurosci.* 8:2. 10.3389/fnsyn.2016.00002 26903855 PMC4744840

[B41] Ting J. T. Daigle T. L. Chen Q. Feng G. (2014). Acute brain slice methods for adult and aging animals: application of targeted patch clamp analysis and optogenetics. *Methods Mol. Biol.* 1183 221–242. 10.1007/978-1-4939-1096-0_14 25023312 PMC4219416

[B42] Trudeau L. E. Emery D. G. Haydon P. G. (1996). Direct modulation of the secretory machinery underlies PKA-dependent synaptic facilitation in hippocampal neurons. *Neuron* 17 789–797. 10.1016/s0896-6273(00)80210-x 8893035

[B43] Wan Q. Xiong Z. G. Man H. Y. Ackerley C. A. Braunton J. Lu W. Y. (1997). Recruitment of functional GABA(A) receptors to postsynaptic domains by insulin. *Nature* 388 686–690. 9262404 10.1038/41792

[B44] Wang W. Wang Q. Qi X. Gurney M. Perry G. Volkow N. D. (2024). Associations of semaglutide with first-time diagnosis of Alzheimer’s disease in patients with type 2 diabetes: target trial emulation using nationwide real-world data in the US. *Alzheimers Dement*. 20 8661–8672. 10.1002/alz.14313 39445596 PMC11667504

[B45] Westermark P. Wernstedt C. Wilander E. Sletten K. (1986). A novel peptide in the calcitonin gene related peptide family as an amyloid fibril protein in the endocrine pancreas. *Biochem. Biophys. Res. Commun.* 140 827–831. 10.1016/0006-291x(86)90708-4 3535798

[B46] Yamashita M. Kawaguchi S. Y. Hori T. Takahashi T. (2018). Vesicular GABA uptake can be rate limiting for recovery of IPSCs from synaptic depression. *Cell Rep.* 22 3134–3141. 10.1016/j.celrep.2018.02.080 29562170

[B47] Zhang Y. Song W. (2017). Islet amyloid polypeptide: another key molecule in Alzheimer’s pathogenesis? *Prog. Neurobiol.* 153 100–120. 28274676 10.1016/j.pneurobio.2017.03.001

