# Supplementary material for: Rodent islet amyloid polypeptide (IAPP) selectively enhances GABAA receptor-mediated neuronal inhibition in mouse ventral but not dorsal hippocampal dentate gyrus granule cells
Source: Front Cell Neurosci. 2025 Feb 19;19:1531790. doi: 10.3389/fncel.2025.1531790 (PMC11880208; doi:10.3389/fncel.2025.1531790)
Supplement: Supplementary file 1 [file Data_Sheet_1.docx]

**Supplementary Figure 1. The effect of rodent islet amyloid polypeptide (rIAPP) on spontaneous synaptic GABA_A_ receptor-mediated currents in the dentate gyrus (DG) granule cells in dorsal and ventral mouse hippocampus.**

Representative continuous traces of spontaneous postsynaptic inhibitory currents (sIPSCs) recorded from DG granule cells in dorsal (DH, **Aa., Ab.**) and ventral (VH, **Ba., Bb.**) hippocampus under control conditions (ACSF) and bath rIAPP (10 pM) application. Marked regions with filled squares (**Aa., Ba.**) are shown on an expanded scale (**Ab., Bb.**).

**
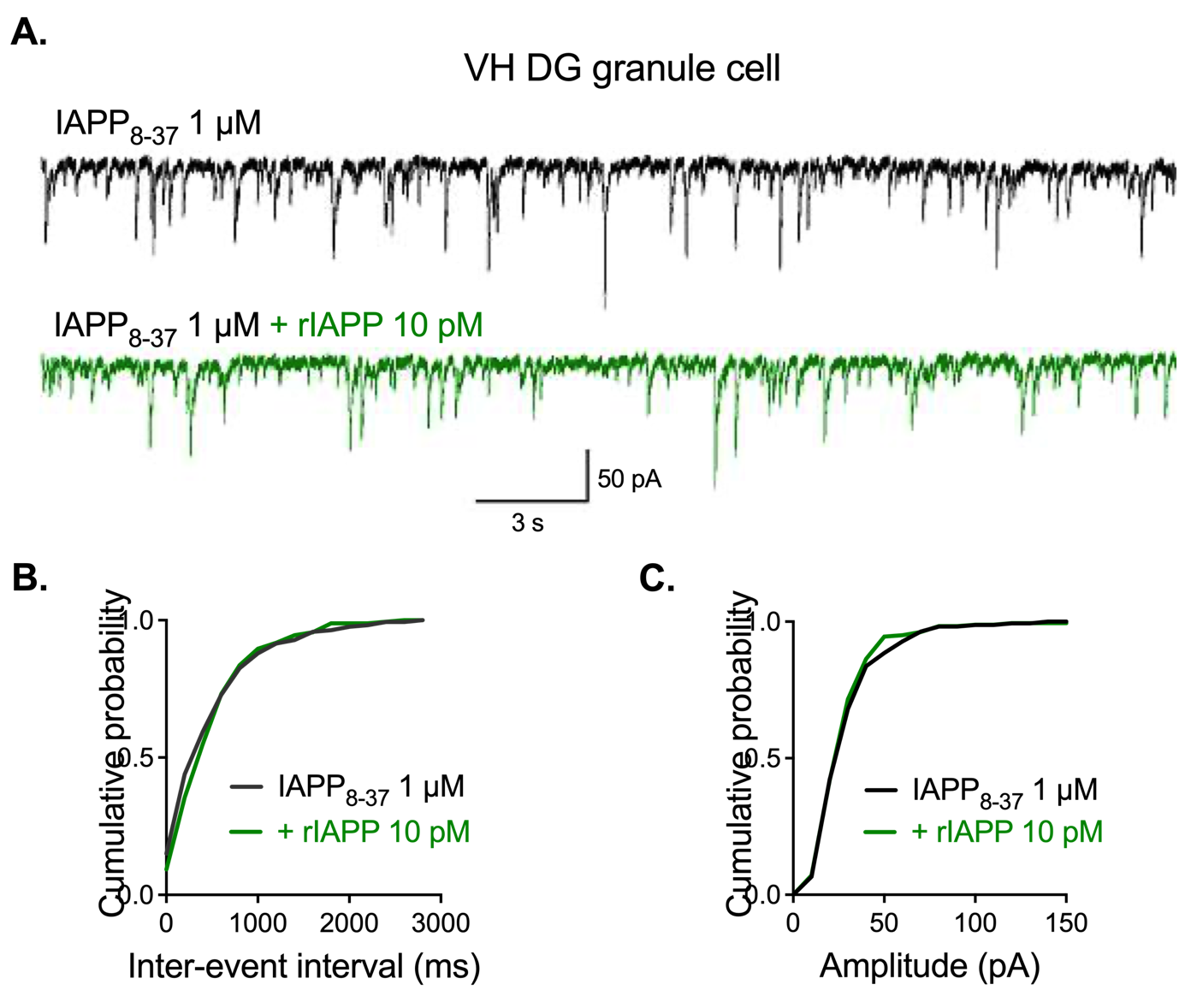
**

**Supplementary Figure 2. IAPP_8-37_ eliminates the effect of rIAPP on synaptic currents in DG granule cells of the ventral hippocampus.**

**(A.)** Representative traces of GABAergic currents recorded from DG granule cells in ventral hippocampus (VH) in constant presence of the 8–37 fragment of Islet Amyloid Polypeptide (IAPP_8-37_, AMYR antagonist, 1 μM) before *(black trace)* and during rIAPP (10 pM) application *(green trace).* There were no differences in cumulative probability distribution plots of inter-event intervals (**B.**) and amplitude (**C.**) values of sIPSCs recorded from DG granule cells of ventral hippocampus in the presence of IAPP_8-37_ (*black*; 166 events analysed for IAPP_8-37_) and during rIAPP co-application (*green*; 183 events analysed for rIAPP and IAPP_8-37_ co-application). V_hold_ = - 60 mV.

**Supplementary Figure 3. The effect of rIAPP on miniature synaptic GABA_A_ receptor-mediated currents in the dentate gyrus (DG) granule cells in dorsal and ventral mouse hippocampus.**

Representative continuous traces of miniature postsynaptic inhibitory currents (mIPSCs) recorded from DG granule cells in dorsal (DH, **Aa., Ab.**) and ventral (VH, **Ba., Bb.**) hippocampus in the presence of TTX (1µM) under rIAPP (10 pM) application. Marked regions with filled squares (**Aa., Ba.**) are shown on an expanded scale (**Ab., Bb.**).

**Supplementary Table 1. rIAPP effect on GABA-mediated IPSC parameters in the dorsal and ventral hippocampal DG granule cells.**

|  | sIPSC | | | |  | mIPSC | | | |
| --- | --- | --- | --- | --- | --- | --- | --- | --- | --- |
|  | DH (n=5) | | VH (n=9) | |  | DH (n=6) | | VH (n=10) | |
|  | ACSF | +rIAPP | ACSF | +rIAPP |  | TTX | +rIAPP | TTX | +rIAPP |
| Rise time 10-90% (ms) | 1.56 ± 0.18 | 1.62 ± 0.17 | 1.86 ± 0.20 | 1.83 ± 0.16 |  | 1.77 ± 0.19 | 1.79 ± 0.22 | 1.51 ± 0.11 | 1.56 ± 0.13 |
| Decay time 63% (ms) | 14 ± 1.5 | 15.55 ± 1.9 | 15.41 ± 1.17 | 15.31 ± 1.03 |  | 16.66 ± 2.25 | 17.63 ± 2.58 | 14.72 ± 1.22 | 15.6 ± 1.07 |
| Charge transfer (fC) | 230.7 ± 15.48 | 235.2 ± 18.36 | 247.9 ± 16.85 | 261.1 ± 16.85 |  | 250.5 ± 22.18 | 264.4 ± 32.05 | 211.3 ± 17.38 | 230.4 ± 16.15* |

The paired student’s t-test was used for comparing values before- and during rIAPP application. *, P < 0.05.
